# Correlations of mRNA Levels among Efflux Transporters, Transcriptional Regulators, and Scaffold Proteins in Non-Small-Cell Lung Cancer

**DOI:** 10.1155/2021/4005327

**Published:** 2021-11-28

**Authors:** Xieyi Zhang, Wangyang Liu, Kazue Edaki, Yuta Nakazawa, Hiroki Kamioka, Atsushi Fujita, Ryoichi Onozato, Misa Iijima, Shigeru Tsuchida, Takahiro Arai, Yukiyoshi Fujita, Kenta Mizoi, Takuo Ogihara

**Affiliations:** ^1^Laboratory of Biopharmaceutics, Department of Pharmacology, Faculty of Pharmacy, Takasaki University of Health and Welfare, 60 Nakaorui-chou, Takasaki-shi, Gunma 370-0033, Japan; ^2^Laboratory of Clinical Pharmacokinetics, Graduate School of Pharmaceutical Sciences, Takasaki University of Health and Welfare, 60 Nakaorui-chou, Takasaki-shi, Gunma 370-0033, Japan; ^3^Department of General Thoracic Surgery, Gunma Prefectural Cancer Center, 617-1 Takahayashinishi-chou, Ota-shi, Gunma 373-0828, Japan; ^4^Department of Pathology and Clinical Laboratories, Gunma Prefectural Cancer Center, 617-1 Takahayashinishi-chou, Ota-shi, Gunma 373-0828, Japan; ^5^Division of Clinical Laboratory, Gunma Prefectural Cancer Center, 617-1 Takahayashinishi-chou, Ota-shi, Gunma 373-0828, Japan; ^6^Division of Pharmacy, Gunma Prefectural Cancer Center, 617-1 Takahayashinishi-chou, Ota-shi, Gunma 373-0828, Japan

## Abstract

Multidrug resistance (MDR) due to enhanced drug efflux activity of tumor cells can severely impact the efficacy of antitumor therapies. We recently showed that increased activity of the efflux transporter P-glycoprotein (P-gp) associated with activation of Snail transcriptional regulators may be mediated mainly by moesin in lung cancer cells. Here, we aimed to systematically evaluate the relationships among mRNA expression levels of efflux transporters (P-gp, breast cancer resistance protein (BCRP), and multidrug resistance-associated protein 2 (MRP2)), scaffold proteins (ezrin (Ezr), radixin (Rdx), and moesin (Msn); ERM proteins), and SNAI family members (Snail, Slug, and Smac) in clinical lung cancer and noncancer samples. We found high correlations between relative (cancer/noncancer) mRNA expression levels of Snail and Msn, Msn and P-gp, Slug and MRP2, and Smuc and BCRP. These findings support our previous conclusion that Snail regulates P-gp activity via Msn and further suggest that Slug and Smuc may contribute to the functional regulation of MRP2 and BCRP, respectively, in lung cancer cells. This trial is registered with UMIN000023923.

## 1. Introduction

Lung cancer is the leading cause of cancer death in the United States. Indeed, almost one-quarter of all cancer deaths are due to lung cancer [1], and among patients with non-small-cell lung cancer (NSCLC), only 26% are alive for more than 5 years after diagnosis [2]. In many patients, surgical operation is not feasible, and thus, chemotherapy and adjuvant therapy are used as first-line treatments [3]. However, these treatments are often adversely affected by the emergence of multidrug resistance (MDR), i.e., resistance to multiple mechanistically and structurally unrelated antitumor drugs [4], resulting in a poor prognosis [5].

A major cause of MDR is increased activity of drug efflux transporters, such as P-glycoprotein (P-gp, ATP-binding cassette subfamily B member (ABCB1)), breast cancer resistance protein (BCRP, ATP-binding cassette subfamily G member 2 (ABCG2)), and multidrug resistance-associated protein 2 (MRP2, ATP-binding cassette subfamily C member 2 (ABCC2)), leading to reduced accumulation of anticancer drugs in cells [6]. P-gp was first identified as being associated with MDR in leukemia patients [7]. However, P-gp is also present in normal tissues, where it has a protective role, mediating the efflux transport of xenobiotics. P-gp functions as an efflux pump for a wide range of amphiphilic, bulky type II cationic drugs and other hydrophobic compounds, including endogenous and exogenous metabolites or toxins, steroid hormones, hydrophobic peptides, and even glycolipids [8]. BCRP has been suggested to be closely associated with resistance of NSCLC to topoisomerase I inhibitors, including irinotecan and its active metabolite, SN-38 [9], while MRP2 contributes to cisplatin resistance [10]. Recently, scaffold proteins, including ezrin (Ezr), radixin (Rdx), and moesin (Msn) (ERM proteins), have been shown to regulate the location of efflux transporters at the cell membrane, consequently regulating their functional activity [6]. They appear to serve as couriers to deliver the transporter molecules to the cell membrane [11–13]. In HCC827 human lung adenocarcinoma cells, Ezr or Msn knockdown significantly reduced Rhodamine123 (Rho123) efflux, which suggests that Ezr and/or Msn are involved in the regulation of P-gp activity in the lung [14].

Members of the SNAI family, including Snail (SNAI1), Slug (SNAI2), and Smuc (SNAI3), are considered as indicators of the malignancy of cancer [15]. Increased malignancy with loss of cell-cell adhesion is associated with epithelial-mesenchymal transition (EMT), which is the first step in the conversion of primary epithelial cells into mesenchymal cells [16]. EMT is associated with altered expression of various genes [17], and SNAI family members are transcriptional regulators that trigger EMT [18]. A positive correlation between Snail and BCRP has been found in breast cancer [19]. However, a recent study indicated that BCRP function was decreased via a reduction of the expression level in HCC827 lung cancer cells with Snail-induced EMT [20]. We previously found that Snail-induced EMT increased the membrane localization and activity of MRP5, but not those of MRP2, in HCC827 cells, resulting in increased cisplatin resistance [20]. We also showed that a histone deacetylase inhibitor, entinostat, suppresses not only Snail-induced cancer malignant alteration, but also P-gp-mediated MDR [21]. Moreover, Snail induces ERM proteins [14]. Transfection of lung cancer cells with Snail reduced the expression levels of epithelial markers, whereas expression of mesenchymal markers was increased [22]. Furthermore, the mRNA expression level of Msn, but not Ezr or Rdx, was increased [12]. Overexpression of Snail induced an increase in P-gp activity in HCC827 cells, and the increase in P-gp activity was suppressed by knockdown of Msn [12]. Thus, Snail may regulate Msn expression and thereby regulate the efflux activity of P-gp in the lung cancer HCC827 cell line [12, 22]. Moreover, these two studies confirmed that the mRNA and protein expression levels of P-gp were consistent in both Mock- and Snail-overexpressing cells.

Although the relationships among transcription regulators, efflux transporters, and ERM proteins at the cellular level have been investigated to some extent, the clinically relevant associations are unknown [6]. Therefore, we set out to systematically evaluate the relationships among efflux transporters (P-gp, BCRP, and MRP2), ERM proteins, and SNAI family members in clinical samples of cancer and noncancer lung tissues from the same subjects in order to identify cancer-related changes. In the present work, we focused on mRNA expression levels in order to identify efflux transporter-regulatory mechanisms operating at the transcriptional level.

## 2. Materials and Methods

### 2.1. Clinical Samples

Surgically excised lung tissues, including cancer tissues and adjacent noncancer tissues, were obtained from 9 patients between 2016 and 2018 in the Gunma Prefectural Cancer Center (Gunma, Japan). Informed consent was obtained from each patient, and studies were performed with the approval of the Ethics Committee of Takasaki University of Health and Welfare (approval no. #2906) and Gunma Prefectural Cancer Center (approval no. #405-29042), in accordance with the Declaration of Helsinki. The study was preregistered in the University Hospital Medical Information Network (UMIN) clinical trial system (UMIN000023923). The characteristics of the nine patients included in the current study are summarized in Table 1. The histopathological subtype was adenocarcinoma (AC) in five patients and squamous-cell carcinoma in four patients.

### 2.2. mRNA Extraction and cDNA Synthesis

mRNA was extracted from NSCLC cell lines seeded on 24-well culture plates using the reported method [23]. Lung tissue (about 200 mg) from patients was initially homogenized using a disposable homogenizer (Nippi, Tokyo, Japan). Total RNA was extracted using a NucleoSpin RNA Midi (Machery-Nagel, Düren, Germany) according to the manufacturer's instructions. cDNA was synthesized from 1 mg total RNA with ReverTraAce (Toyobo, Osaka, Japan) according to the manufacturer's instructions, using a T100™ Thermal Cycler.

### 2.3. Quantitative Real-Time Polymerase Chain Reaction (qRT-PCR)

Quantitation of mRNA expression level by qRT-PCR was performed with Power SYBER™ Green PCR Master Mix (Applied Biosystems, CA, USA) using the MX3000P™ Multiplex Quantitative PCR System (Stratagene, CA, USA). The mRNA expression levels of target genes were quantified relative to that of the housekeeping gene GAPDH according to the following equation (Ct: threshold line):

(1) $$\begin{array}{c} \text{gene expression level}=1000\times 0.5^{-\left(Ct\left(\text{GAPDH}\right)-Ct\left(\text{target gene}\right)\right)}. \end{array}$$

The absolute value was defined as the gene expression level in the cancer tissue. The relative value was defined as the ratio of the gene expression level in the cancer tissue to that in the noncancer tissue.

### 2.4. Statistical Analysis

All results are presented as mean ± standard deviation (SD). Pearson's test or the Spearman correlation test was used to obtain correlation coefficients. The criterion of statistical significance was *p* < 0.05.

## 3. Results

### 3.1. Correlations of mRNA Expression Levels in Cancer Tissues (Absolute Values)

**Figure 1:**
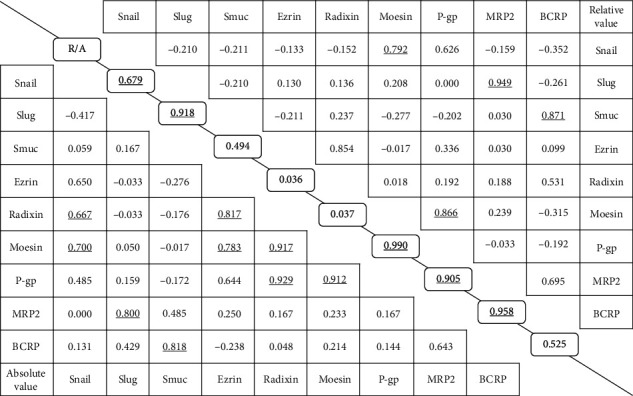
Correlations (*r* values) among absolute and relative mRNA expression levels of SNAI family members, ERM proteins, and efflux transporters in clinical lung cancer tissue samples. Each value in the part below the oblique line shows the correlation of the absolute values of the parameters in the corresponding row and column, and those in the part above the oblique line show the correlation of the relative values of the parameters in the corresponding row and column. The values on the oblique line show the correlation between relative and absolute values (R/A) of the same parameter. The boldface underline indicates *p* *<* *0.05*.

Expression levels of Snail mRNA were highly correlated with Rdx (*r* = 0.667) and Msn (*r* = 0.700) in lung cancer tissues. Slug mRNA levels showed a high correlation with MRP2 (*r* = 0.800), and Smuc mRNA levels showed a high correlation with BCRP (*r* = 0.818) (Figure 1).

Expression levels of Rdx and Msn mRNAs showed very high correlations with P-gp (*r* = 0.929 for Rdx and *r* = 0.912 for Msn). In addition, ERM protein expression levels were highly correlated with each other (Ezr vs. Rdx, *r* = 0.817; Ezr vs. Msn, *r* = 0.783; Rdx vs. Msn, *r* = 0.917).

### 3.2. Correlations of mRNA Expression Levels in Cancer and Noncancer Tissues (Relative Values)

**Figure 2:**
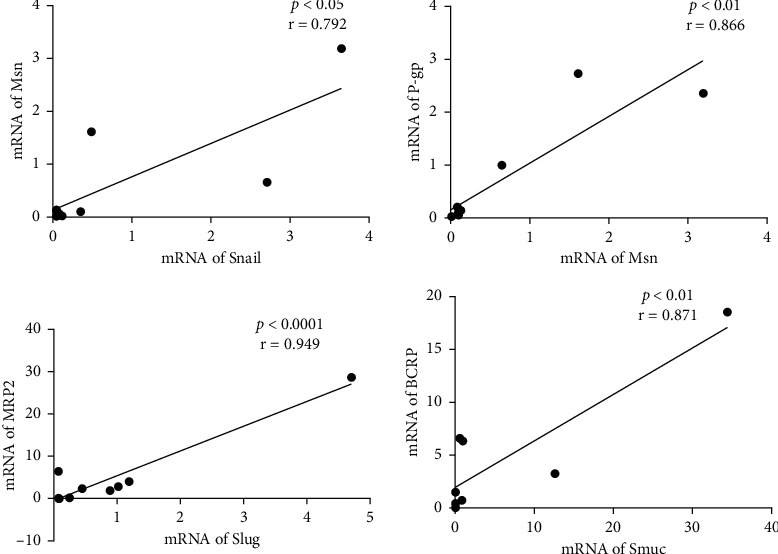
Correlations of relative mRNA expression levels of Snail and Msn, Msn and P-gp, Slug and MRP2, and Smuc and BCRP.

The relative (cancer/noncancer) mRNA expression values showed a high correlation between Snail and Msn (*r* = 0.792) and also between Msn and P-gp (*r* = 0.866). Slug and MRP2 showed an extremely high correlation (*r* = 0.949), and Smuc and BCRP were also highly correlated (*r* = 0.871) (Figure 2). However, in contrast to the absolute values, significant correlations were not found between Rdx and Snail or between Rdx and P-gp. There was also no correlation among ERM proteins.

### 3.3. Correlations between Relative and Absolute Values of mRNA Expression

The relative and absolute values were highly correlated for Snail (*r* = 0.679), Slug (*r* = 0.918), Msn (*r* = 0.990), P-gp (*r* = 0.905), and MRP2 (*r* = 0.958), but not for Smuc, Ezr, Rdx, or BCRP.

## 4. Discussion

mRNA expression level is often used as an indicator of protein expression and function [24]. In our previous study, we reported that Snail-overexpressing HCC827 cells showed significantly increased mRNA expression of Msn compared with Mock cells, whereas the expression levels of Ezr and Rdx were unchanged [12]. The protein expression level of Msn was consistent with the mRNA expression level. Moreover, Msn knockdown blocks the increase in P-gp activity in HCC827 cells. Independently, we have also found that P-gp function is regulated by the ERM proteins. Our present findings are consistent with that report. We also confirmed a high correlation of relative values of mRNA expression levels between Snail with Msn in NSCLC.

We further investigated whether the mRNA expression levels of ERM proteins were correlated with that of P-gp. As regards relative values, only Msn was correlated with P-gp mRNA expression. However, we previously found that, in HCC827 cells, Ezr or Msn knockdown significantly reduced the Rho123 efflux rate, suggesting that Ezr and/or Msn are involved in the membrane expression of P-gp in the lung [14]. This is reasonable, since it is well known that mRNA expression levels of transporters do not necessarily correlate with protein expression level or functional activity [25]. Moreover, P-gp function in clinical samples may change depending on factors such as the age and disease status of the patients.

Rdx regulates MRP2 function in the liver, both in normal tissues and in cancer cells [26]. Reduced phosphorylation of threonine at the C-terminal of Rdx impairs the binding of MRP2 to actin, which decreases the localization of MRP2 to the cell membrane [27]. BCRP function was evaluated using NSCLC HCC827 cells and was found to be correlated with expression of Ezr and Msn [14]. Here, we found that the mRNA expression of MRP2 was positively correlated with that of Slug, and the mRNA expression of BCRP was positively correlated with that of Smuc, for both absolute and relative values. However, no relationship was observed between MRP2/BCRP and ERM proteins. Our results suggest that Slug and Smuc may contribute to the functional regulation of MRP2 and BCRP, respectively, in lung cancer patients.

There are several limitations in the present study. Firstly, we used only clinical samples, because it is difficult to acquire the lung tissue from normal subjects. Secondly, we measured mRNA expression levels of efflux transporters, which may be useful to identify regulatory mechanisms operating at the transcriptional level, although it should be noted that they are not necessarily reflected in changes of protein expression or functional activity. Further studies using lung cancer cell lines are underway to address these issues. Moreover, only nine patients, mostly with early-stage disease based on their TNM classifications, were included in this study. Further clinical samples, especially from patients with advanced metastatic tumors, are needed to confirm our findings.

## 5. Conclusions

In conclusion, high correlations were found between the relative mRNA expression levels of Snail and Msn and Msn and P-gp, suggesting that the Snail/Msn pathway contributes to the functional regulation of P-gp. Our findings also suggest that Slug and Smuc may contribute to the functional regulation of MRP2 and BCRP, respectively, in lung cancer tissues.

## Figures and Tables

**Table 1:** Characteristics of patients from whom samples were obtained.

Patient no.	Gender	Age (years)	Histological subtype	Stage	TNM classification
1	Female	63	AC	IA	T1aN0M0
2	Male	72	AC	IA	T1bN0M0
3	Male	75	SCC	IIA	T2aN1M0
4	Female	71	AC	IA	T1bN0M0
5	Male	63	SCC	IIIA	T2bN2M0
6	Male	76	SCC	IB	T2aN0M0
7	Female	72	AC	IA2	T1bN0M0
8	Male	68	AC	IB	T2aN0M0
9	Male	76	SCC	IB	T2aN0M0

AC: adenocarcinoma; SCC: squamous-cell carcinoma.

## Data Availability

The data used to support the findings of this study are included within the article.
